# Contemporary Polymer-Based Nanoparticle Systems for Photothermal Therapy

**DOI:** 10.3390/polym10121357

**Published:** 2018-12-07

**Authors:** Jeremy B. Vines, Dong-Jin Lim, Hansoo Park

**Affiliations:** 1 Organogenesis, Surgical and Sports Medicine, Birmingham, AL 35216, USA; jbvines@gmail.com; 2 Otolaryngology Head & Neck Surgery, University of Alabama at Birmingham, Birmingham, AL 35294, USA; 3 School of Integrative Engineering, Chung-Ang University, Seoul 06974, Korea

**Keywords:** photothermal therapy, polymeric nanoparticle, polyaniline, polypyrrole, polydopamine

## Abstract

Current approaches for the treatment of cancer, such as chemotherapy, radiotherapy, immunotherapy, and surgery, are limited by various factors, such as inadvertent necrosis of healthy cells, immunological destruction, or secondary cancer development. Hyperthermic therapy is a promising strategy intended to mitigate many of the shortcomings associated with traditional therapeutic approaches. However, to utilize this approach effectively, it must be targeted to specific tumor sites to prevent adverse side effects. In this regard, photothermal therapy, using intravenously-administered nanoparticle materials capable of eliciting hyperthermic effects in combination with the precise application of light in the near-infrared spectrum, has shown promise. Many different materials have been proposed, including various inorganic materials such as Au, Ag, and Germanium, and C-based materials. Unfortunately, these materials are limited by concerns about accumulation and potential cytotoxicity. Polymer-based nanoparticle systems have been investigated to overcome limitations associated with traditional inorganic nanoparticle systems. Some of the materials that have been investigated for this purpose include polypyrrole, poly-(3,4-ethylenedioxythiophene):poly(4-styrenesulfonate) (PEDOT:PSS), polydopamine, and polyaniline. The purpose of this review is to summarize these contemporary polymer-based nanoparticle technologies to acquire an understanding of their current applications and explore the potential for future improvements.

## 1. Introduction

In 2017, cancer was the second-most common cause of death in the United States, comprising 22.5% of the total number of deaths; 591,699 people died from complications related to cancer in 2017 [1]. Unfortunately, owing to the heterogenous nature of cancer, there are currently no fully comprehensive approaches for treatment; options are mainly limited to chemotherapy, radiotherapy, immunotherapy, and surgery. Although these approaches provide some therapeutic efficacy, they are limited by their risk to normal, healthy cells, their potential to destroy the immune system, or by conferring an increased risk for the development of secondary cancers [2,3,4].

Owing to the limitations currently associated with standard cancer treatment modalities, the focus of research has shifted to alternative techniques that may mitigate the limitations associated with traditional cancer therapies. One of these treatment modalities is known as hyperthermic therapy. Hyperthermia, the state of elevated body temperature, has been thoroughly explored for cancer therapy. In this process, cancer cells are subjected to thermal stress, which induces apoptosis. In clinical settings, hyperthermic therapy is utilized either alone or in a combinational approach with radiotherapy (RT) or chemotherapy (CT) [5]. For example, when clinically-relevant drugs for malignant melanoma are combined with low or high hyperthermia (43 °C and 45 °C, respectively), intrinsic or extrinsic ER-mediated apoptosis was induced [6].

This was first demonstrated when human T-cell acute lymphoblastic leukemia cell lines were used to show the bimodality of hyperthermia and RT. It was shown that radiation-induced cancer cell death could be enhanced [7]. The mechanism of this synergism comes from the potential heat-induced inhibition of DNA repair; the heat prevented the cancer cells from restoring their viability after exposure to radiation-induced DNA damage [8].

To date, there have been many preclinical studies confirming that hyperthermia can enhance RT or chemoradiotherapy (RTCT) [9]. However, conventional HT techniques are not considered to be ideal as they are not tumor-focused, are not minimally invasive, and create a uniform generation of heat throughout the body [10]. These factors lead to substantial undesirable side effects. For example, whole-body hyperthermia may cause gastrointestinal symptoms and cardiovascular side effects [11]. Hence, nanoparticle-mediated localized hyperthermia would be a more promising modality for cancer treatment. One such promising treatment modality that continues to gain attention and is currently under investigation for potential widespread use is photothermal therapy (PTT) [12,13]. PTT is based on the principle of the conversion of light energy (usually in the near-infrared region) into heat energy, which then induces cellular necrosis or apoptosis [14].

Compared with other methods, light is an ideal external stimulus as it is easily regulated, focused, and remotely controlled. This ease of focus and control enable better targeted treatments that lead to less damage to healthy tissues [15,16,17]. However, photodynamic therapy of tissues mediated by either laser or visible light is limited by the depth of penetration of the light [18,19,20]. However, near-infrared (NIR) light (in the wavelength range of 800–1200 nm) has much greater body transparency and is preferred for photothermal therapy. In contrast to photodynamic therapy (PDT), which relies on the presence of oxygen to generate reactive oxygen species, and is considerably limited in application due to its limited depth of penetration [20], PTT mainly exerts effects by increasing the local temperature within tumors [21]. Regarding this, it has been demonstrated that in order to completely destroy cancer cells in vitro, a threshold temperature ranging between 70 °C and 80 °C is required [22]. Furthermore, at temperatures ranging from 55 to 95 °C, tumorigenic damage is evident within in vivo conditions [23].

To be considered an ideal candidate for PTT, a specific set of requirements should be met. For example, the ideal PTT candidate should be: (i) of a suitable nanoparticulate size and of uniform shape; (ii) have good dispersibility in aqueous solutions; (iii) respond to light in the NIR range of 650–950 nm to prevent damage to surrounding healthy tissues, provide sufficient photothermal efficiency, and to enable sufficient depth of penetration; (iv) be sufficiently photostable to ensure adequate diffusion time to reach tumors before losing their photosensitivity, (v) exhibit low or no cytotoxicity in living systems [24].

Currently, the available PTT-enabling agents mainly comprise metal nanoparticles (gold (Au) [25,26,27,28,29], silver (Ag) [30,31,32], palladium [33,34], and germanium (Ge) [35], semiconducting nanoparticles [36,37,38], and carbon-based nanomaterials (carbon nanotubes and graphenes) [36,39,40,41,42,43,44,45,46]. A majority of the currently available PTT-enabling nanoparticle technologies use inorganic photothermal agents, sometimes consisting of heavy metals, which are non-biodegradable and may remain in the body for long periods, thus precluding their utilization for long-term cancer therapies owing to accumulation and potential toxicity concerns. Although many inorganic nanoparticle-based materials, such as gold and carbon, have shown initial promise in both in vivo and in vitro studies, their potential long-term toxicity is as an obstacle to immediate clinical translation [41,46,47,48,49]. For example, it is known that carbon-based nanomaterials may induce many toxic responses, such as pulmonary inflammation and the generation of reactive oxygen species [50,51].

Alternatively, polymeric materials geared towards applications in PTT have attracted much attention. To date, polypyrrole, poly-(3,4-ethylenedioxythiophene):poly(4-styrenesulfonate) (PEDOT:PSS), dopamine-melanin (polydopamine), and polyaniline nanoparticles have been reported to show photothermal effects [52,53,54,55] (Table 1). The interest in these materials has grown as they are typically considered superior on account of their enhanced biodegradability and biocompatibility in conjunction with considerable photothermal efficiency. Therefore, in this review, we have explored the commonly investigated polymer-based nanoparticle systems intended for photothermal therapy to determine their potential applications in treatment.

## 2. Polyaniline-Based Systems

**Figure 1:**
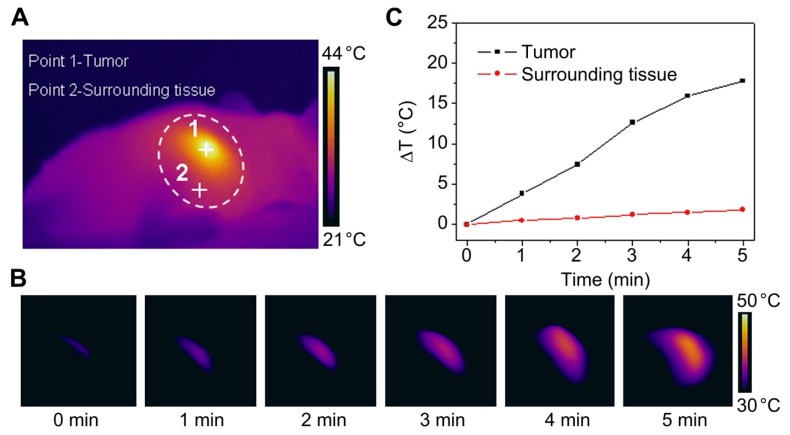
An example of polyaniline-based nanoparticles for photothermal therapy. (**A**) Photothermal images of a mouse after intratumoral injection of F127-modified polyaniline nanoparticles (F-PANPs) (1 mg/mL) with an 808 nm laser (0.5 W cm^−2^, 3 min). When irradiated within the white circle area, the tumor (Point 1) and the surrounding tissue (Point 2) were heated. (**B**) Photothermal images of the tumor obtained at different time points. The scale bar indicates the temperature difference between 30 °C and 50 °C. (**C**) The relationship between sample temperature and laser illumination time is shown in (**A**). Reproduced from [24] with permission, copyright Elsevier, 2013.

Among the polymeric materials discussed in this review, polyaniline is perhaps one of the oldest conducting polymers utilized for PTT (Figure 1) [24]. This material is consistently recognized for its low cost, good conductivity, and mechanical flexibility [77]. In addition, polyaniline is known to be non-cytotoxic and was used as an electroactive tissue for studying cellular proliferation prior to its utilization in PTT [78].

The utilization of polyaniline for photothermal ablation of cancer cells was first proposed by Huh et al. in 2011 [56]. In this study, the synthesis of polyaniline nanoparticles was achieved by doping polyaniline-based nanoparticles via a chemical oxidation polymerization method. Using this synthesis method, polyaniline nanoparticles were obtained in the form of an emeraldine salt. These particles exhibited strong NIR absorption, making them viable for potential PTT applications. Although this was an important leap, the synthesized polyaniline nanoparticles were relatively large, with a mean diameter of 115.6 nm. In addition, although their photothermal capabilities were assessed in in vitro experiments, their in vivo applications in PTT were not investigated.

Therefore, to rectify the issues regarding size and to assess their in vivo photothermal capabilities, Zhou et. al synthesized polyaniline nanoparticles of a suitable size and uniform shape by using an environmentally-friendly hydrothermal method; nanoparticle sizes of 48.5 ± 1.5 nm were achieved [24]. The nanoparticles were further functionalized by capping with F-127 surfactant (F-PANPs) to increase their hydrophilic conversion. NIR photothermal conversion efficiencies and molar extinction coefficients were first determined, followed by the in vitro and in vivo assessment of the photothermal efficacy of the nanoparticles. In vitro assessments using HCT116 cancer cells demonstrated increased apoptosis via NIR-mediated photothermal induction and in vivo experiments using mice bearing HCT116 xenograft tumors revealed photothermal-mediated tumor suppression. Biochemical indicators for hepatic and kidney functions were measured from the blood serum collections and were found to be within the normal ranges, indicating that the nanoparticle injections did not result in any noticeable cytotoxicity.

Multifunctional nanoparticles were developed to facilitate photodynamic and photothermal therapy, and consisted of indocyanine green loaded onto PEGylated nanoparticles containing a silver nanoparticle core with a polyaniline shell (ICG-Ag@PANI) [57]. The combination of polyaniline and indocyanine green led to a synergistic effect in which the hyperthermal efficacy of the nanoparticles was enhanced, allowing rapid reactive oxygen species (ROS) generation via NIR light-mediated temperature increases to 56.8 °C within 5 min. The ability of these nanoparticles to elicit cytotoxic effects in HeLa cells was demonstrated in vitro and in vivo studies demonstrated tumor growth inhibition.

Multifunctional polyaniline-based nanoparticles were created through the synthesis of polyaniline with methotrexate (MTX), a well-known chemotherapeutic agent [58]. To enhance the tumor-selective targeting and uptake, lanreotide (LT), a synthetic analog of somatostatin, was conjugated to the surface of the nanoparticles (LT-MTX/PANI NPs) to target the somatostatin surface receptors, which are overexpressed in many cancer cells. The multifunctional capabilities of these nanoparticle systems were supported by the enhanced apoptosis enabled by the heat-associated drug release of MTX in response to NIR. Enhanced tumor targeting and uptake were also found in xenograft tumor-bearing mice and led to the improved suppression of tumor development, when compared with MTX, PANI NPs, or MTX/PANI NPs alone.

To allow selective targeting and to enhance biodegradability and tumor uptake, polyaniline nanoparticles were first coated with a lipid layer and then conjugated with folic acid (FA-Lipid-PANI NPs) [59]. The lipid-coated polyaniline showed enhanced biodegradability, which reduced cytotoxicity and enhanced tumor permeability. The conjugation with folic acid enabled active tumor targeting through folic receptor-mediated transcytosis. In vitro experiments with HeLa cells and in vivo experiments with BALB/c mice expressing HeLa-induced tumors demonstrated that the nanoparticles could elicit NIR light-mediated cellular necrosis and apoptosis both in vitro and in vivo, as well as selective tumor targeting and suppression in vivo.

Multifunctional nanoparticles were created in which WS_2_ nanodots were coated with polyaniline shells [60]. In this configuration. WS_2_ nanodots conferred radiosensitivity and polyaniline provided the hyperthermic capability. Subsequently, the shells were loaded with hyaluronic acid and chlorin e6 (Ce6) to provide selective tumor targeting via HA and photodynamic capability via Ce6. The multi-functional particles exhibited enhanced tumor uptake and were capable of facilitating multi-modal imaging (PA, CT, and FL). Additionally in vitro and in vivo experiments demonstrated photothermal capability, as illustrated by the fact that the particles exhibited photothermal effects both in vitro and in vivo, with no signs of cytotoxicity in vivo.

In a recent study, multifunctional nanoparticles capable of selective tumor targeting and photothermal effects were created via the encapsulation of polyaniline and cisplastin within folate-poly (ethylene glycol)-distearoylphosphatidylcholine (FA-PEG-DSPE), cRGD [cyclic (Arg-Gly-Asp-D-Phe-Lys)]-PEG-DSPE, and lecithin conjugates dubbed FA/cRGD-PNPs [61]. The synthesis procedure led to nanoparticles with a uniform size of 102.7 nm and an enhanced apoptosis rate in MDA-MB-231 cells compared with chemotherapy or photothermal treatment alone, to achieve a cellular death rate of approximately 92.6% after 24 h of treatment.

## 3. Polypyrrole-Based Systems 

**Figure 2:**
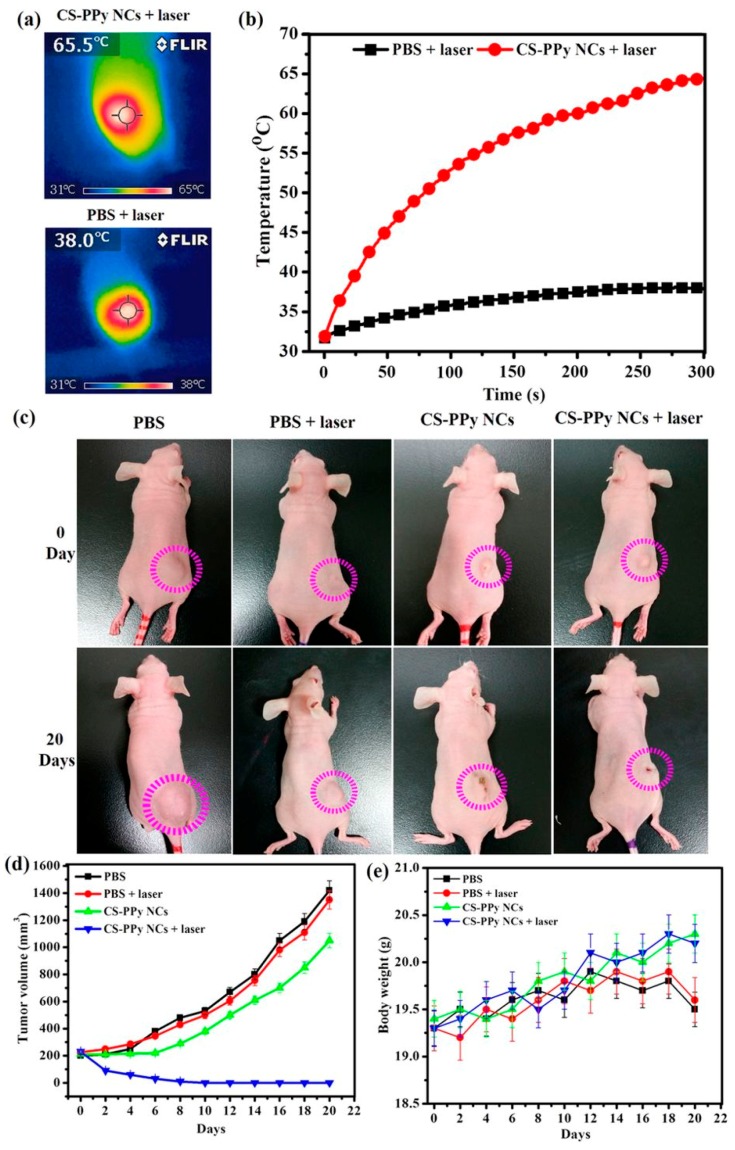
A polypyrrole-based nanocomposite for near-infrared (NIR) photothermal therapy. NIR thermographic images (**a**) and temperature change (**b**) in tumor-bearing mice after intratumoral injection of PBS and chitosan-polypyrrole nanocomposites (CS-PPy NCs) and irradiation with an 808 nm laser (2.0 W cm^−2^, 5 min). (**c**) The change in tumor size between Day 0 (before treatment) and 20 days after treatment. (**d**) Tumor volume growth curves for different groups of mice after different treatments. (**e**) The body weight after different treatments indicated over 20 days. The error bars indicate the mean ± standard deviation. Reproduced from [80] under open access license.

Polypyrrole (PPy) serves as one of the most commonly utilized base materials for use in hyperthermia-based PTT cancer treatments (Figure 2) [79,80]. PPy was first documented in the early 20th century, where it was known as “pyrrole black”, as it was a black precipitate from acidic pyrrole/H_2_O_2_ aqueous solutions. PPy has recently found popularity as an electroresponsive material in biomedical engineering applications [81,82,83] as it is generally regarded as biocompatible, with little or no adverse effect on health [84,85].

Since its initial discovery, various methods, such as electrochemical synthesis, have been explored for the synthesis of PPy. The most common method of PPy synthesis is through an oxidative reaction in which pyrrole is polymerized via a pseudo-polycondensation mechanism [86,87]. In this process, pyrrole is first converted to a radical cation via a one-electron oxidation, after which two pyrrole radical cations couple to form 2,2′-bipyrrole, which can then couple with yet another radical cation. This sequence is then repeated to form longer chains. This technique is highly convenient, as it can be completed chemically by using a chemical oxidant in solution (e.g., (NH_4_)_2_S_2_O_8_, FeCl_3_, H_2_O_2_) [88,89,90] or electrochemically by using an electrode to apply an oxidizing potential [91,92]. The end morphology of PPy is strongly dependent on the synthesis conditions and methodology used. Most current synthesis methods result in the production of bulk configurations of PPy, such as sheets; however, to be feasible for in vivo cancer therapies, particulates between 30 nm and 300 nm should be created.

In many cases, PPy NPs have been shown to exhibit higher photothermal conversion efficiencies than even Au nanorods, which are well-known photothermal agents [62]. PPy NPs with an average size of ~50 nm were prepared using aqueous dispersion polymerization with FeCl_3_ as an oxidation agent. PVP was then used as a capping agent. The synthesized PPy NPs demonstrated a strong NIR absorption peak at approximately 850 nm [55]. These NPs were highly biocompatible, inducing no negative effects on QSG-7701 human hepatocyte cells up to 200 ppm. To demonstrate the photothermal efficiency of these PPy NPs, an in vivo NT1 tumor model was constructed in BALB/c mice.

PPy NPs with an average diameter of 46 nm were created [62]. In this study, PPy NPs had no cytotoxic effect on human umbilical vein endothelial cells (HUVECs), but induced the death of HeLa cells in combination with NIR irradiation, which demonstrated their ability to elicit targeted cell death via hyperthermia.

Much like other nanomaterials, PPy-based therapeutic platforms can be further functionalized by loading with anti-tumorigenic medications (chemotherapeutics) or conjugated with tumor-specific antibodies to enhance their specificity compared with other nanoparticles. An injectable system of hollow microspheres as developed to produce localized heat upon stimulation with NIR light to mediate antibiotic release [93].

Inorganic materials may also be used to serve as intermediary molecules to allow the conjugation of other functional units. For example, mesoporous amino-functionalized dendrimer-like silica nanoparticles (DSNs-NH_2_) were created via the hydrolysis of tetraethyl orthosilicate (TEOS) and 3-aminopropyl-triethoxysilane (APTES) in a special emulsion to form nanoparticles with a 233 nm diameter [63]. PPy chains were then formed on the nanoparticles. To further improve biocompatibility, the resultant PPy@DSNs-NH_2_ nanoparticles were conjugated with poly(ethylene glycol) monomethyl ether at the carboxyl group (PEG-COOH) to obtain PEG. The nanoparticles were then loaded with doxorubicin (DOX) to enable a dual-acting therapeutic approach wherein either pH or NIR light could trigger DOX release, thus combining a chemotherapeutic approach with a hyperthermic approach provided by the presence of PPy.

Owing to the flexibility of PPy synthesis, PPy drug carriers with different morphologies may be developed by using different shaped templates. In one study, spindle-like polypyrrole hollow nanocapsules (PPy HNCs) were created by using spindle-like Fe_2_O_3_ particles as a template [64]. By loading DOX within these hollow PPy nanoparticles, more effective cell penetration was enabled in comparison with spherical PPy nanoparticles of the same size (~155 nm). This hybrid chemo- and photothermal platform was demonstrated to be effective in a HepG2 tumor model in BALB/c nude mice.

It was also shown that PPy could be combined with other functional platforms to improve tumor specificity and efficacy. For example, PPy and the chemotherapeutic agent rapamycin were loaded into liposomes conjugated with trastuzumab (a monoclonal antibody targeting the HER2/neu receptor) to create a combined chemo-photothermal therapy system (LRPmAB) [65]. This system showed enhanced uptake in BT-474 cells, whereas cells that did not overexpress the HER2/neu receptors showed comparatively lower uptake.

## 4. Polydopamine-Based Systems

As previously discussed, polymeric-based nanomaterials have been investigated for many years. However, the degradation properties of many of these polymers are still not fully understood; hence, there is the potential for unknown adverse effects. However, polydopamine, a natural occurring polymer, has established biodegradation properties that do not result in adverse effects [94].

Polydopamine nanoparticles were first explored as a potential photothermal agent by Liu et al. [53]. They created dopamine–melanin colloidal nanospheres through the oxidation and self-polymerization of dopamine in a mixture containing water, ethanol, and ammonia at room temperature. Melanin, a naturally occurring biopolymer, is known to have many functions in living organisms, including thermoregulation and protection from ultraviolet injury [95]. To perform these functions, the absorption of melanin can extend into the NIR region. By tuning the molar ratio of ammonia to dopamine, the size of the fabricated nanoparticles could be controlled: an increase from 11.3 to 17, created dopamine-melanin nanoparticles of ~70 nm. To confirm the hyperthermic capability of these nanoparticles was tested in vitro with HeLa and 4T1 cells along with in vivo testing in BALB/c mice exhibiting 4T1 tumors. The results demonstrated tumor reduction and continued tumor suppression. Furthermore, serum biochemistry analysis of five hepatic indicators revealed results within the normal ranges, which demonstrated that the injection did not cause any systematic toxicity.

However, while PDA nanoparticles exhibited initial promise, their mass-extinction co-efficient in the NIR region was relatively low, making less than ideal for phototherapy. Owing to this limitation, a study was conducted in which PDA nanoparticles were synthesized via the oxidation-induced self-polymerization of dopamine in an alkaline environment [66]. The nanoparticles were then PEGylated to enhance their physiological stability and conjugated with ICG to shift the absorbance peak from 780 nm to 800 nm (PDA-ICG-PEG). By taking advantage of π-π stacking and hydrophobic interactions, the chemotherapeutic drug doxorubicin was able to be loaded to the nanoparticles at 150% loading capacity (DOX/PDA *w*/*w*). Prior to this study, chemotherapeutic approaches had not been explored in combination with photothermal approaches using PDA.

As with many materials, PEGylation is a commonly employed modification that is utilized to enhance tumorigenic targeting and uptake. Polydopamine is no exception, and PEGylation has been employed to polydopamine nanoparticles in other studies as well for this same reason [67]. In this same study, anticancer drugs, such as 7-ethyl-10-hydroxycampthotheticin (SN38), were loaded onto the PDA-PEG nanoparticles via π-π interactions. Owing to the nature of these interactions, the drug compounds remained conjugated at physiological conditions until they were released in an on-demand fashion following exposure to NIR light. In vivo experiments demonstrated that nanoparticles loaded with SN38 suppressed tumor growth via the combined approach of chemo- and photothermal therapies.

In another study employing a similar combinatorial approach, a novel core-shell nanoparticle system consisting of doxorubicin (Dox-M) encapsulated within DSPE-PEG micelles was developed and then coated with polydopamine [68]. The monoclonal antibody bortezomib (Btz), which has an affinity for proteasomes associated with many cancers, was conjugated to polydopamine to enhance targeting and efficacy of the therapy (Dox-M@PDA-Btz). By combining Dox, Btz, and PDA into a single nanoplatform, an enhanced chemo-photothermal therapy approach was created, allowing for NIR mediated hyperthemic therapies along with pH-mediated release of chemotherapeutics for the treatment of breast cancer.

Polydopamine has been used in a few instances to enhance the biocompatibility of and confer hyperthermic functionality to inorganic substances for a multifunctional approach. For example, a couple of studies coated iron oxide nanoparticles with polydopamine to create multifunctional particles capable of magnetic field targeting for improved photothermal cancer therapy [69,70]. In other studies, pre-existing photothermal substances, such as gold nanorods, were coated with polydopamine to provide synergistic enhancements to their photothermal efficiency [71,72]. Therefore, the surface modification with polydopamine is recognized as a good strategy to enhance the biocompatibility of non-biodegradable substances [96].

## 5. Poly(3,4-ethylenedioxythiophene):poly(4-styrenesulfonate) (PEDOT:PSS)

Poly(3,4-ethylenedioxythiophene):poly(4-styrenesulfonate) (PEDOT:PSS) is another class of polymer-based nanoparticles commonly used for NIR-mediated hyperthermic therapy.

In perhaps the first documented study of this material for photothermal cancer therapy, PEGylated PEDOT:PSS nanoparticles (PDOT:PSS-PEG) were synthesized via a layer-by-layer approach, creating nanoparticles of approximately 80 nm in diameter [52]. Owing to the inherent properties of this material, a long blood circulation half-life of 21.4 ± 3.1 h was found, which permitted considerable accumulation of the particles within tumors owing to the EPR effect. Experiments with mice bearing 4T1 tumors demonstrated the ability of these PDOT:PSS-PEG nanoparticles to eliminate tumors via NIR-mediated photothermal ablation. In addition, blood panel and serum biochemistry tests conducted at 7 and 40 days post-injection were within normal parameters, which demonstrated that the nanoparticles were not cytotoxic in vivo.

In a follow-up study, PEDOT:PSS nanoparticles were conjugated with other molecules for a multifunctional approach [73]. Following initial PEGylation (PEDOT:PSS-PEG), the chemotherapeutic drugs doxorubicin and SN38, as well as Ce6, were loaded onto the nanoparticles via hydrophobic interactions and *π*-*π* stacking. Unexpectedly, it was also shown that the solubility of water-insoluble drugs, such as SN38, can be enhanced by loading the PEDOT:PSS-PEG nanoparticles while maintaining cytotoxicity. Through the combination of chemotherapeutic drugs and photothermal enhancers such as Ce6, synergism was established, which improved the therapeutic efficacy.

In a recent study, tri-modal nanoparticles were fabricated by using a combination of magnetic nanoparticles, PEDOT:PSS, Cyanine7 (Cy7), and 2-deoxyglucose (2-DG)-polyethylene glycol (MNP@PES-Cy7/2-DG) to combine nanomagnetic-based hyperthermia with NIR fluorescence. Additionally, owing to the inclusion of 2-deoxyglucose, a glucose analog, nanoparticle uptake was enhanced for more selective targeting of tumor cells [74]. This has been demonstrated to occur in other polymeric-based hyperthermic systems, as PEDOT:PSS was used to simultaneously enhance the biocompatibility of, and confer hypothermic ability to, inorganic materials, such as iron oxide, to enable simultaneous multimodal image-guided hyperthermia [75].

## 6. Other Polymer-Based Nanoparticle Systems and Future Approaches

Although the majority of polymer-based photothermal therapy systems are based on polypyrrhole, polyaniline, or PEDOT:PSS, novel polymer-based nanomaterial systems for PTT have continued to emerge, displaying enhanced functionality.

**Figure 3:**
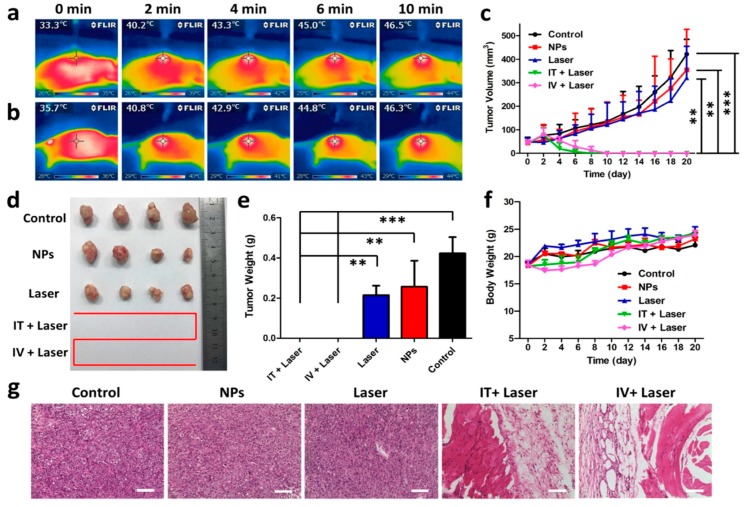
The NIR-II photothermal approach by a novel nanoagent. In vivo photothermal therapy of tumors. Mice were subjected to whole-body IR images after injection with a narrow band gap D–A conjugated polymer (TBDOPV–DT), with 2,2-bithiophene as the donor and thiophene-fused benzodifurandione-based oligo(*p*-phenylenevinylene) as the acceptor. (**a**) Intratumoral (0.56 mg kg^−1^, 0.9 W cm^−2^, and a 1064 nm laser) or (**b**) intravenous (1.94 mg kg^−1^, 1.3 W cm^−2^) administration. (**c**) Growth rates of tumors after different treatments. (**d**) Representative size of the excised tumors. (**e**) Weight of tumor in each condition. (**f**) Changes in body weight of the mice with tumors. ** *p* ≤ 0.01; *** *p* ≤ 0.001. (**g**) H&E staining of tumor regions in different groups. Scale bars indicate 100 μm. Reproduced from [76] with permission, copyright American Chemical Society, 2018.

NIR light at wavelengths of 750–1000 nm (NIR-I) has been typically used in PTT as it enables much greater depth of tissue penetration than light in the visible spectrum [97,98,99,100,101,102,103]. However, there has been growing interest in even deeper NIR light (NIR-II), which involves the utilization of NIR light of wavelengths of 1000–1700 nm (Figure 3) [76]. The advantage of utilizing light in this range is even deeper tissue penetration compared with light in the NIR-I range and a higher maximum permissible exposure to lasers [104,105,106,107].

Taking this fact into consideration, Xie et al. developed a novel NIR-II photothermal nanoagent by functionalizing a narrow band gap D-A conjugated polymer (TBDOPV-DT), with 2,2-bithiophene serving as a donor and thiophene-fused benzodifurandione-based oligo(*p*-phenylenevinylene) as an acceptor (TBDOPV-DT NPs) [76]. By utilizing this novel nanoparticle system that is responsive to light in the NIR-II range, a much greater depth of tissue penetration was achieved, exhibiting greater temperature elevations at up to 8 mm of depth compared with NIR-I-responsive nanomaterials. In vivo studies of HeLa xenograft tumor-bearing mice demonstrated that the nanoparticle system was capable of eliciting considerable tumor suppression. Serum biochemistry assessments for liver and kidney functional markers fell within normal ranges, demonstrating a lack of apparent cytotoxicity.

Therefore, the development of polymers capable of responding to light in the NIR-II range will enable enhanced therapeutic outcomes and should be considered in the future development of polymer-based nanoparticle systems. As discussed in this review, several polymeric nanoparticle systems have been demonstrated to be activated by the NIR spectrum, providing sufficient energy to exert hyperthermic effects. However, compared with inorganic materials, the performance of polymeric nanoparticles is likely to be weak because of photobleaching effects. To overcome this innate drawback, more sophisticated photothermal therapy based on polymeric nanoparticle systems should be pursed. Improved antitumor response by advanced NIR-triggered drug release might be able to overcome the natural pitfalls of polymeric-based nanoparticle systems.

## 7. Conclusions

Cancer is recognized as the second major cause of death in the United States. Unfortunately, traditional methods for the treatment of cancer, such as chemotherapy, radiotherapy, immunotherapy, and surgery are plagued by limitations, which either resulting in incomplete tumor removal or the induction of undesirable side effects. Materials such as polypyrrole, polyaniline, polydopamine, and PEDOT:PSS have proven to be flexible platforms with the capability of multifunctional modifications that enabling synergistic approaches with traditional cancer therapies, potentially mitigating any adverse side effects. These technologies have shown great promise, and new technologies with enhanced capabilities, such as those enabling PTT via the utilization of light in the NIR-II range, thereby achieving greater depth of penetration have started to emerge.

## Figures and Tables

**Table 1:** Summary of Polymer Based Nanoparticle Systems for Photothermal Therapy.

Polymer	Configuration	Testing Stage	Reference
Polyaniline	Nanoparticle	In-Vitro	[56]
	F-127 Conjugated Nanoparticle	In-Vitro and In-Vivo	[24]
	Silver nanoparticle core, polyaniline shell (ICG-Ag@PANI)	In-Vitro and In-Vivo	[57]
	Nanoparticles with lanreotide and methotrexate (LT-MTX/PANI NPs)	In-Vitro and In-Vivo	[58]
	Nanoparticles with lipid layer and folic acid conjugation (FA-Lipid-PANI NPs)	In-Vitro and In-Vivo	[59]
	WS core, polyaniline shell with hyaluronic acid and clorin e6 (Ce6)	In-Vitro and In-Vivo	[60]
	polyaniline and cisplastin within folate-poly (ethylene glycol)-distearoylphosphatidylcholine (FA-PEG-DSPE), cRGD [cyclic (Arg-Gly-Asp-D-Phe-Lys)]-PEG-DSPE, and lecithin conjugates dubbed FA/cRGD-PNPs	In-Vitro and In-Vivo	[61]
Polypyrrole	Base Nanoparticles	In-Vitro and In-Vivo	[55]
	Base Nanoparticles	In-Vitro	[62]
	Polypyrrole chains synthesized on mesoporous amno-functionalized dendrimer-like silica nanoparticles (DSNs-NH2) PPy@DSNs-NH2 loaded with doxorubicin	In-Vitro	[63]
	Spindle-like hollow polypyrrole nanocapsules (PPy HNCs) loaded with doxorubicin	In-Vivo	[64]
	Ppy and rapamycin loaded into liposomes conjugated with trastuzumab (LRPmAB)	In-Vitro	[65]
Polydopamine	Dopamine-melanin colloidal nanospheres	In-Vitro and In-Vivo	[53]
	PEGYlated polydopamine nanoparticles conjugatd with ICG (PDA-ICG-PEG) loaded with doxorubicin	In-Vitro	[66]
	PEGylated nanoparticles loaded with 7-ethyl-10-hydroxycampthotheticin (SN38)	In-Vivo	[67]
	Doxorubicin encapsulated within DSPE-PEG micelles coated with polydopamine	In-Vitro and In-Vivo	[68]
	Fe(3)O(4) core polydopamine coated nanoshell	In-Vitro	[69,70]
	polydopamine coated gold nanorods	In-Vitro	[71]
	Polydopamine coated gold/silver nanoparticles	In-Vitro	[72]
PEDOT:PSS	PEGylated PEDOT:PSS nanoparticles (PDOT:PSS-PEG)	In-Vivo	[52]
	PEDOT:PSS-PEG loaded with doxorubicin, SN38, and Ce6	In-Vitro	[73]
	Magnetic nanoparticles with PEDOT:PSS, Cyanine7 (Cy7), and 2-deoxyglucose (2-DG)-polyethylene glcol (MNP@PES-Cy7/2-DG)	In-Vitro and In-Vivo	[74]
	Magnetic nanoparticles with PEDOT:PSS coating	In-Vivo	[75]
TBDOPV-DT	D-A conjugated polymer (TBDOPV-DT), with 2,2-bithiophene serving as a donor and thiophene-fused benzodifurandione-based oligo(p-phenylenevinylene) as an acceptor (TBDOPV-DT NPs)	In-Vitro and In-Vivo	[76]
